# Association among presence of cancer pain, inadequate pain control, and psychotropic drug use

**DOI:** 10.1371/journal.pone.0178742

**Published:** 2017-06-08

**Authors:** Paula Parás-Bravo, María Paz-Zulueta, María Cristina Alonso-Blanco, Paloma Salvadores-Fuentes, Ana Rosa Alconero-Camarero, Miguel Santibañez

**Affiliations:** 1 Department of Nursing, University of Cantabria, Santander, Spain; 2 Department of Nursing, University “Rey Juan Carlos”, Madrid, Spain; 3 Department of Physical Therapy, Occupational Therapy, Rehabilitation and Physical Medicine, Division of Physical Therapy, University “Rey Juan Carlos”, Madrid, Spain; University of the Chinese Academy of Sciences, CHINA

## Abstract

**Introduction:**

Pain is a common symptom in cancer patients, and its control and management are complex. Despite the high concomitant use of psychotropic drugs among such patients, the association among pain, inadequate pain control, and psychotropic drug use has not been fully determined. This study examined the prevalence of cancer pain and inadequate pain control and the association with psychotropic drug use.

**Materials and methods:**

In this cross-sectional study, we investigated 402 medical records obtained by simple random sampling of oncology patients at a hospital in northern Spain from July 2012 to July 2014. Adjusted odds ratios (ORs) were estimated together with their 95% confidence intervals (95% CIs) by unconditional logistic regression for each type of psychotropic drug (anxiolytics, hypnotics, and antidepressants).

**Results:**

The mean patient age was 61.17 (standard deviation ± 13.14) years; 57.5% were women, 42.5% men. Pain was present in 18.4% of patients and inadequate pain control in 54.2%. We found a statistically significant association between the presence of cancer pain and anxiolytic use (adjusted OR, 3.15; 95% CI, 1.49–6.68) and hypnotic use (adjusted OR, 5.19; 95% CI, 1.77–15.25). Inadequate pain control was associated to a greater extent with the use of those drugs: adjusted OR for anxiolytic use, 4.74 (95% CI, 1.91–11.80); adjusted OR for hypnotic use, 6.09 (95% CI, 1.74–21.32). By contrast, no association was found between pain and antidepressant use (adjusted OR, 0.99).

**Conclusion:**

The presence of pain and (to a greater extent) poor pain control were associated with increased use of certain psychotropic drugs, such as anxiolytics and hypnotics. There appeared to be no association between pain and antidepressant use.

## Introduction

The International Association for the Study of Pain defines pain as an ‘unpleasant sensory and emotional experience associated with actual or potential tissue damage, or described in terms of such damage’ [1]. There is no universally accepted definition for ‘cancer pain’; however, a high percentage of cancer patients experience pain that often cannot be controlled [2–4]. According to estimates, 20%–50% of cancer patients suffer from pain [5]; the proportion rises to 90% in advanced stages [6]. The origin of pain is variable: it may be due to the disease itself, aspects derived from treatment, or associated comorbidities [7]. A review of 46 studies published between 1987 and 2007 reported that on average, pain was undertreated in 43.4% of cancer patients; however, the figure could be as high as 82% [4]. A later review reported that 31.8% of patients had inadequately controlled pain, with poor control rising to almost 70% [3].

Pain is thus a fundamental aspect in the treatment of cancer patients. That point was established at the 58th World Health Assembly on Cancer Prevention and Control of the World Health Organization (WHO). The assembly concluded that pain relief and palliative care should be prioritized in world cancer programmes; it urged health-care systems to establish programmes for monitoring pain control [8].

The treatment and control of cancer pain is complex; authorities struggle to reach consensus regarding the treatment of choice [7,9]. The European Oncology Nursing Society proposes re-evaluating patients to ensure the efficacy of treatment [7,10]; individualized treatment for each patient is of course recommended [2]. Since 1986, the WHO has considered psychotropic drugs to be an adjunctive treatment for pain in cancer patients [11]. In their guidelines on managing cancer pain, the American Cancer Society [12] and European Society for Medical Oncology [13] currently include the use of psychotropic drugs to help relieve pain (antidepressants and benzodiazepines). Psychotropic drugs may enhance the effect of pain medicines (or lessen their side effects)—especially in the case of refractory pain. Accordingly, optimal pain control could be associated with the use of these medicines.

Psychotropic drugs are commonly used in combination with analgesics in oncological patients. In a study of women with breast cancer and pain, Syrowatka et al. [14] reported consumption of 50.6% and 22.4%, respectively, of anxiolytics and antidepressants during the course of active treatment. In a sample of patients with metastatic cancer and pain, Barry et al. [15] observed anxiolytic and antidepressant consumption, respectively, in 51% and 64% of cases. The authors found that 38% of the patients consumed three or more psychotropic drugs. Together with the basal situation of the patient, this consumption of analgesics and psychotropic drugs could increase the appearance of adverse effects. It is therefore recommended that such patients be carefully monitored [16–18]; it may be necessary to replace the psychotropic drugs with psychological or behavioural interventions [14].

Around one-third of cancer patients are estimated to experience some type of mental health problem during the course of active treatment [17,18]. The complex oncological process occasionally causes patients to suffer major emotional distress [19–21]: symptoms of anxiety (20%) and depression (13%) predominate; they are sometimes accompanied by physical symptoms, such as insomnia and headache [22,23]. This leads to increased use of psychotropic drugs.

Emotional distress in cancer patients is usually associated with pain [21,24]. Consequently, the use of psychotropic drugs may also be related to the presence of pain or be exacerbated by poor pain control. However, the effect of pain or poor pain control on symptoms (anxiety, depression, and insomnia) and psychotropic drug use is not fully established. Accordingly, the aim of the present study was to determine the prevalence of cancer pain and inadequate pain control in cancer patients; the study also analysed the association among pain, poor pain control, and psychotropic drug use.

## Materials and methods

The study protocol was approved by the Cantabria Clinical Research Ethics Committee on 29 May 2014; that was before data acquisition, which began in February 2015. The relevant authorization was also obtained in line with hospital regulations. The data were treated anonymously and confidentially under Spanish Organic Law 15/1999 of 13 December on Personal Data Protection. Because the data were analysed anonymously, it was not necessary to obtain the consent of patients to investigate their data. This study received the 16th National Nursing Research Award Valdecilla, which funds social work in Caja Cantabria (Spain). The funders had no role in the study design, data collection and analysis, decision to publish, or preparation of the manuscript.

### Study design and participants

**Fig 1:**
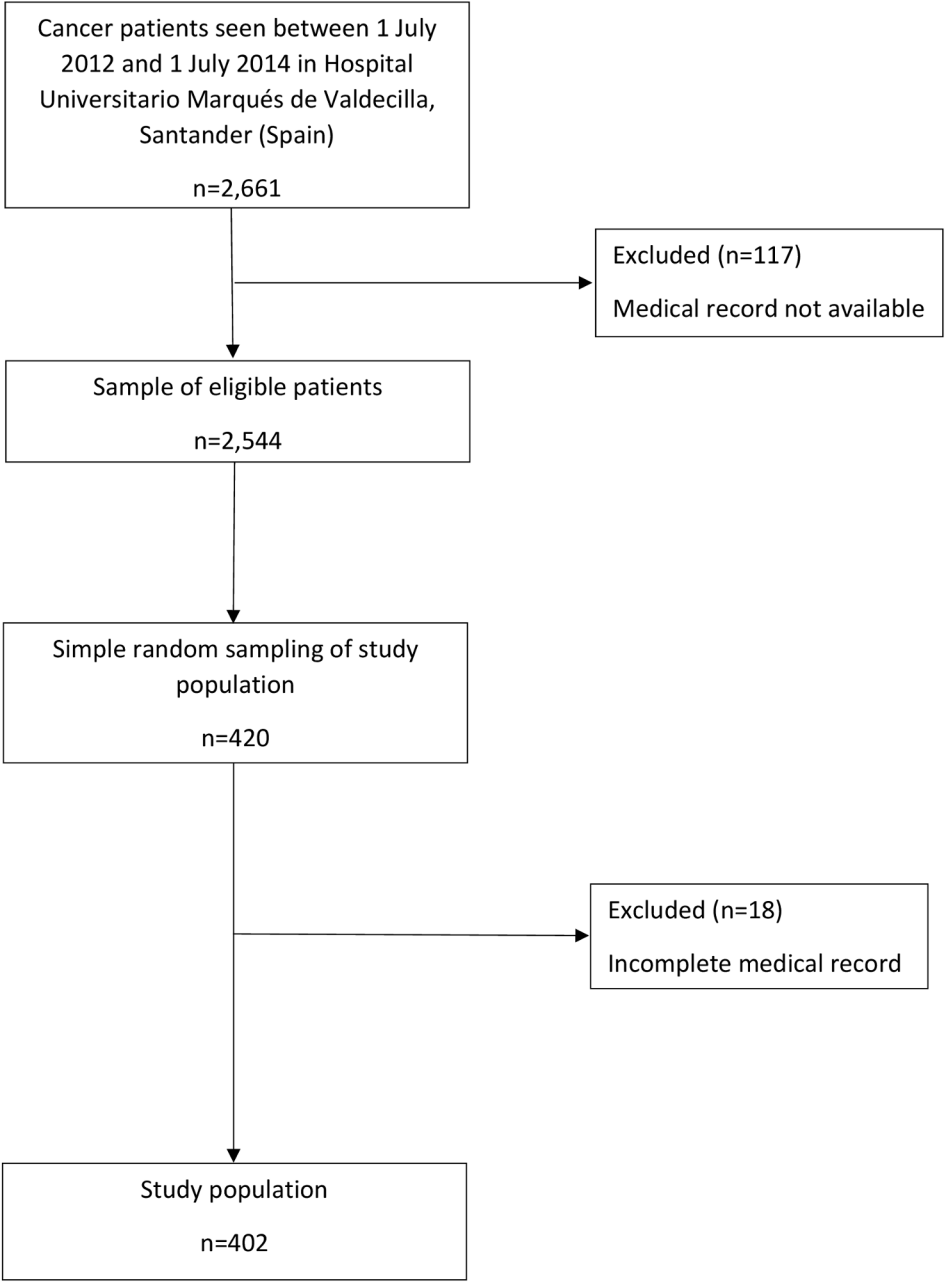
Overall study flow.

In this cross-sectional study, the eligibility criteria were patients aged over 18 years with oncological disease treated at the Oncology Unit of Hospital University Marqués of Valdecilla (National Public Health System of Spain) in northern Spain between 1 July 2012 and 1 July 2014. In all, 420 Oncology Unit patients were selected by simple random sampling. We considered the medical records incomplete when information about all the variables related to pain and the use of each type of psychotropic drug was missing. We considered the records complete if they contained information about >80% of the remaining variables. If the medical records were incomplete, we withdrew the patient from the study. Of the 420 original subjects, we removed 18 patients (4.3%) in this way (Fig 1).

To determine the sample size, we used the following criteria. With respect to prevalence, the literature is scarce, and so we calculated the sample size by assuming maximum indeterminacy (*P* = 0.5). Under that assumption, with a 95% confidence interval for a maximum error of 5%, we estimated a sample of 400 patients. That sample size (n = 400) would have sufficient power (1 – β >80%) to detect associations ≥1.3 as significant for a 50% risk of the effect in the unexposed sample, considering an unexposed/exposed ratio of 1, using a two-tailed chi-square test with an alpha level of 0.05.

### Data sources and variables

We collected data between February and May 2015. From patient medical records and nursing assessments, we obtained information for each cancer patient included who underwent active treatment from 1 July 2012 to 1 July 2014. We obtained full oncological details. We collected the following variables:

- Sociodemographic: age, sex, and education level.
- Family background: marital status and number of children [25].
- Oncological process: type of cancer (location), cancer treatment (chemotherapy, radiotherapy, hormone therapy, biological therapy, surgery), and side effects of cancer treatment. The symptoms considered side effects were those established by the National Cancer Institute [26]. S1 Appendix.
- Pain: presence of pain, location of pain, intensity of pain, and pain control. The presence of pain was treated dichotomously (yes/no). In patients whose pain was treated with analgesics, the type of analgesic, route of administration, and intensity of pain were recorded. The latter was measured following the recommendations of the European Society for Medical Oncology using the Visual Analogue Scale (VAS) [13], from 0 (‘no pain’) to 10 (‘extreme pain’). Self-perceived pain control, reported by the patients themselves, was recorded in the nursing history; this information was obtained directly from the medical record. The variable ‘pain control’ was categorized ordinally into ‘no pain’, ‘adequate pain control’, and ‘inadequate pain control’. The Oncology Unit uses the latest recommendations of the European Society for Medical Oncology to determine the prescription of analgesic treatment. The use of psychotropic drugs was considered according to those recommendations [13]. The cancer pain characteristics of the study population are presented in relation to sex, which may influence pain expression [27,28].
- Use of psychotropic drugs: the use of anxiolytics, hypnotics, and antidepressants was classified dichotomously (yes/no) if a prescription for those treatments (both continuous and on demand) was reported in the patient's medical record.

### Data analysis

The data analysis included an initial descriptive analysis. Means with their standard deviation (SD; or median and interquartile range in the case of asymmetric distributions) were estimated for the continuous variables using the Student *t* test for comparisons or the Mann-Whitney *U* test in the case of non-normal distributions. We estimated proportions for the categorical and discrete variables by means of the Pearson chi-square test, likelihood ratio (LR) chi-square test, Wilcoxon two-sample test, and Fisher exact test as appropriate for comparisons. The use of each type of psychotropic drug was treated as a dependent variable; we performed separate analyses for anxiolytics, hypnotics, and antidepressants. Finally, we calculated a variable that included the joint use of anxiolytics and hypnotics. As a measure of association, we estimated the odds ratios (ORs) together with their 95% confidence intervals (95% CIs) by unconditional logistic regression.

To control for confounding bias in the association between presence of pain, pain control, and use of each psychotropic drug, we predefined the potential confounders. They were as follows: age; sex; marital status; education level; having children; type of cancer (location); chemotherapy; radiotherapy; hormone therapy; biological therapy; surgery; side effects of cancer treatment; and fatigue. We calculated the adjusted ORs for all the predefined potential confounders (maximum model). Furthermore, to obtain better precision in the adjusted estimates, we predefined three additional criteria based on the simulation studies of Mickey and Greenland [29] and Maldonado and Greenland[30]:

1. Associated with the presence or absence of pain with significance levels of *P* ≤0.20.
2. Associated with use of the psychotropic drug under analysis with an OR of 1.5–0.67 as criterion.
3. Causes a major change in the OR when adjusted: a change greater than 10% with the equation (ORc-ORa)/ORa >10% was considered a criterion.

For each psychotropic drug, we constructed an ad hoc optimal sub-model based on fulfilment of the above three criteria. We calculated the following: crude OR (ORc); adjusted OR (ORa) in the maximum model; and adjusted OR in the optimal sub-model.

We set the alpha error at 0.05, and all *P* were bilateral. All statistical analyses were performed using IBM SPSS Statistics, version 22.0.

## Results

Table 1 shows the pain characteristics in the sample of 402 patients according to sex.

**Table 1:** Cancer pain characteristics of the study population related to sex.

			TOTAL (N = 402)		FEMALE		MALE		
					N = 231	57.5%	N = 171	42.5%	
			N	%	N	%	N	%	*p*
**Age (years), Mean [SD]**^**a**^			61.17	[13.14]	60.08	[12.75]	62.64	[13.55]	0.053
**Pain**			** **						
	No		328	81.6	197	85.3	131	76.6	0.026*
	Yes		74	18.4	34	14.7	40	23.4	
**Location of pain**									
	Arthralgias		23	31.1	12	35.3	11	27.5	
	Craniofacial		12	16.2	3	8.8	9	22.5	
	Abdominal		9	12.2	3	8.8	6	15.0	
	Epigastric		8	10.8	4	11.8	4	10.0	
	Lumbar		7	9.5	5	14.7	2	5.0	
	Others		15	29.3	7	20.6	8	20.0	
**VAS**^**b**^**, Mean [SD]**^**a**^			5.64	[1.41]	5.50	[1.28]	5.74	[1.51]	0.621**
**Analgesic use**									
	No		2	2.7	0	0	2	5.0	
	Yes		72	97.3	34	100.0	38	95.0	
**Type of analgesic**									
	Strong Opiods		30	41.7	14	41.2	16	42.1	
	Weak Opiods		4	5.6	2	5.9	2	5.3	
	Non-opoid analgesics		38	52.8	18	25.0	20	27.8	
		Nonsteroidal anti-inflammatory drugs	23	31.9	12	35.3	11	28.9	
		Other Non-opoid analgesics	15	20.8	6	17.6	9	22.5	
**Route of administration**									
	Oral		46	63.9	23	67.6	23	60.5	0.125*
	Transdermal		26	36.1	11	32.4	15	39.5	
**Effectiveness of analgesia**									
	Adequate pain control		33	45.8	17	50	16	42.1	0.108*
	Inadequate pain control		39	54.2	17	50	22	57.9	

^a^ Standard deviation

^b^ Visual Analogue Scale

*Chi-square test

**Wilcoxon two-sample test

The mean patient age was 61.17 (SD = 13.14) years; 57.5% (n = 231) were women and 42.5% (n = 171) were men. Pain was reported by 18.4% (95% CI, 14.5–22.3) of patients (n = 74). Pain was more prevalent in men (23.4% in men, 14.7% in women; *P* = 0.026). With respect to location, arthralgias were the most prevalent in both sexes, being reported by 31.1% of patients (n = 23). The mean pain intensity, measured by the VAS, was 5.64 (SD = 1.41) points; there were no statistically significant differences between the sexes (*P* = 0.621).

Among the 74 patients who reported pain, treatment was with analgesics in 72 (97.3%). With respect to the type of analgesic, strong opioids were prescribed in 41.7% (n = 30) and weak opioids in 5.6% (n = 4). Non-opioid analgesics were prescribed in 52.8% of patients (n = 38). The most common route of administration was oral, which used in 63.9% of cases (n = 46). With respect to pain control, among the 72 patients treated with analgesics, 45.8% (95% CI, 33.6–58.0) (n = 33) reported adequate pain control, whereas 54.2% (95% CI, 42.0–66.4) (n = 39) reported inadequate pain control; there were no statistically significant differences with sex (*P* = 0.108).

Table 2 shows the use of psychotropic drugs according to the presence of pain. In all, 40.5% (95% CI, 28.7–52.4) (n = 30) of patients with pain reported taking some type of psychotropic drug. With respect to the type of psychotropic drug, 32.4% used anxiolytics, 12.2% hypnotics, and 4.1% antidepressants. The use of anxiolytics (*P* <0.001) and hypnotics (*P* = 0.004) was significantly higher among the patients with pain. Table 2 shows the potential confounders according to the presence of pain.

**Table 2:** Factors associated with presence of cancer pain.

				TOTAL (N = 402)		EXISTENCE OF CANCER PAIN				
						No (N = 328)		Yes (N = 74)		
				N	%	N	%	N	%	*p*
**Any psychotropic use**										
	No			321	79.9	277	84.5	44	59.5	<0.001*
	Yes			81	20.2	51	15.5	30	40.5	
**Anxiolytic use**										
	No			334	83.1	284	86.6	50	67.6	<0.001*
	Yes			68	16.9	44	13.4	24	32.4	
**Type of anxiolytic**										
	Long-acting benzodiazepines			22	32.4	18	40.9	4	16.7	0.041*
	Short-acting benzodiazepines			46	67.7	26	59.1	20	83.3	
**Hypnotic use**										
	No			384	95.5	319	97.3	65	87.8	0.004***
	Yes			18	4.5	9	2.7	9	12.2	
**Type of hypnotic**										
	Benzodiazepines			9	50.0	5	55.6	4	44.4	>0.005****
	Other			9	50.0	4	44.4	5	55.6	
**Antidepressant use**										
	No			385	95.8	314	95.7	71	96.0	0.934*
	Yes			17	4.2	14	4.3	3	4.1	
**Type of antidepresant**										
	SSRIs ^a^			17	100.0	14	100.0	3	100.0	
**Potential confounders**										
	Gender									
		Female		231	57.5	197	60.1	34	46.0	0.027*
		Male		171	42.5	131	39.9	40	54.1	
	Age (year), mean [SD] ^b^			61.17	[13.14]	61.14	[13.50]	61.30	[11.53]	0.845**
	Marital status									
		Married		276	77.7	229	79.5	47	70.1	0.150*
		Single		50	14.1	40	13.9	10	14.9	
		Divorced/Separated		11	3.0	7	2.4	4	6.0	
		Widowed		18	5.1	12	4.2	6	9.0	
		Missing		47	11.7	40	12.2	7	9.5	
	Children									
		No		88	24.8	69	24.0	19	28.4	
		Yes		267	75.2	219	76.0	48	71.6	0.457*
		Missing		47	11.7	40	12.2	7	9.5	
	Number of children, mean [SD] ^b^			1.83	[1.01]	1.82	[1.04]	1.90	[0.93]	0.437**
	Educational level									
		Primary		202	57.5	166	58.2	36	54.5	0.410*
		Secondary		80	22.8	61	21.4	19	28.8	
		University		69	19.7	58	20.4	11	16.7	
		Missing		51	12.7	43	13.1	8	10.8	
	Type of cancer (location)									
		Breast		111	27.6	98	29.9	13	17.6	<0.001*
		Rectal		64	15.9	56	17.1	8	10.8	
		Lung		37	9.2	21	6.4	16	21.6	
		B-cell lymphoma		30	7.5	27	8.2	3	4.1	
		Ovary		14	3.5	12	3.7	2	2.7	
		Other		146	36.3	114	34.8	32	43.2	
	Cancer therapy									
		Chemotherapy								
			No	37	9.2	32	9.8	5	6.8	0.420*
			Yes	365	90.8	296	90.2	69	93.2	
		Radiotherapy								
			No	224	55.7	190	57.9	34	45.9	0.061*
			Yes	178	44.3	138	42.1	40	54.1	
		Hormone therapy								
			No	346	86.1	277	84.5	69	93.2	0.048*
			Yes	56	13.9	51	15.5	5	6.8	
		Biological therapy								
			No	278	69.2	226	68.9	52	70.3	0.818*
			Yes	124	30.8	102	31.1	22	29.7	
		Surgery								
			No	212	52.7	167	50,9	45	60.8	0.124*
			Yes	190	47.3	161	49.1	29	39.2	
	Side effects of cancer treatment									
		No		83	20.6	79	24.1	4	5.4	<0.001*
		Yes		319	79.4	249	75.9	70	94.6	
	Analgesic									
		No		330	82,1	328	100	2	2.7	<0.001*
		Yes		72	17.9	0	0	72	97.3	
	Effectiveness of analgesia									
		Adequate pain control		33	45.8	0	0	33	45.8	
		Inadequate pain control		39	54.2	0	0	39	54.2	
	Fatigue									
		No		206	51.2	165	50.30	41	55.4	0.507*
		Yes		196	48.8	163	49.69	33	44.6	

^a^ SSRIs: selective serotonin reuptake inhibitors

^b^ Standard deviation

*Compared using chi-square test

**Compared using Wilcoxon two-sample test

***Compared using LR chi-square test

****Compared using Fisher exact test

Sex, the type of cancer (location), hormone therapy, and side effects of cancer treatment were statistically significantly associated with the presence of pain. Marital status, radiotherapy, and surgery to remove cancer were associated to a lesser degree, presenting significance levels of *P* ≤0.20. All these variables therefore met the first criterion for being included as confounders in the optimal models.

The association of each of these variables with taking psychotropic drugs can be seen in S1 Table. Table 3 shows the associations among the presence of pain, pain control, and anxiolytic use. The presence of pain was statistically significantly associated with anxiolytic use: ORc, 3.10 (95% CI, 1.7–5.5). This association was strengthened after adjusting for all potential confounders in the maximum model: ORa maximum model, 3.64 (95% CI, 1.6–8.4; S2 Table). The association was also strengthened after the selection of confounders included in the optimum model based on established methodological criteria (sex, marital status, type of cancer [location], and side effects of cancer treatment): ORa optimum model, 3.15 (95% CI, 1.5–6.7). After ordinal categorization based on pain control, the associations showed a statistically significant dose-response pattern: ORc for ‘inadequate pain control’, 4.03 (95% CI, 2.0–8.3; linear *P* trend, <0.001); ORa maximum model, 4.98 (95% CI, 1.8–13.7; linear *P* trend *=* 0.001 S2 Table); and ORa optimum model, 4.74 (95% CI, 1.9–11.8; linear *P* trend = 0.001).

**Table 3:** Association among presence of cancer pain, pain control, and anxiolytic use.

		Anxiolytic use							
		**No (n)**	**Yes (n)**	**ORc**^**a**^	**(95%**	**CI)**	**ORa opt**^**b**^	**(95%**	**CI)**
**Existence of cancer pain**									
	No	284	44	1			1		
	Yes	50	24	3.10	1.7	5.5	3.15	1.5	6.7
** **		**No (n)**	**Yes (n)**	**ORc**^**a**^	**(95%**	**CI)**	**ORa opt**^**b**^	**(95%**	**CI)**
**Pain control**									
	No pain	284	44	1			1		
	Adequate pain control	25	8	2.07	0.9	4.9	1.86	0.6	5.4
	Inadequate pain control	24	15	4.03	2.0	8.3	4.74	1.9	11.8
***Linear p trend***				<0.001			0.001		

^a^ Odds ratios and 95% confidence Intervals. ORc: crude odds ratio

^b^ ORa opt: adjusted OR according to the optimum model (with only selection of confounders included in the regression model according to the criteria described in Materials and Methods (*P* ≤0.20; OR 1.5–0.67, change in OR, >10%): sex, marital status, type of cancer (location), and side effects of cancer treatment

Table 4 shows the associations among presence of pain, pain control, and hypnotic use.

**Table 4:** Association among presence of cancer pain, pain control, and hypnotic use.

		Hypnotic use							
		**No (n)**	**Yes (n)**	**ORc**^**a**^	**(95%**	**CI)**	**ORa opt**^**b**^	**(95%**	**CI)**
**Existence of cancer pain**									
	No	319	9	1			1		
	Yes	65	9	4.91	1.9	12.8	5.19	1.8	15.2
** **		**No (n)**	**Yes (n)**	**ORc**^**a**^	**(95%**	**CI)**	**ORa opt**^**b**^	**(95%**	**CI)**
**Pain control**									
	No pain	319	9	1			1		
	Adequate pain control	29	4	4.89	1.4	16.8	4.26	1.0	17.9
	Inadequate pain control	34	5	5.21	1.6	16.4	6.09	1.7	21.3
***Linear p trend***				0.002			0.003		

^a^ Odds ratio and 95% confidence intervals. ORc: crude odds ratio

^b^ ORa opt: adjusted OR according to the optimum model (with only selection of confounders included in the regression model according to the criteria described in Materials and Methods (*P* ≤0.20; OR 1.5–0.67, change in OR, >10%): marital status and type of cancer (location)

Statistically, the presence of pain was significantly associated with hypnotic use: ORc, 4.91 (95% CI, 1.9–12.8). This association was also strengthened after adjusting for all confounders in the maximum model (ORa maximum model, 5.57; 95% CI, 1.6–19.0; S2 Table) and optimum model (marital status and type of cancer [location]) OR optimum model, 5.19; 95% CI, 1.8–15.3). The association showed a statistically significant dose-response trend: ORc ‘inadequate pain control’, 5.21 (95% CI, 1.6–16.4; linear *P* trend *=* 0.002); ORa maximum model, 7.60 (95% CI, 1.8–31.3; linear *P* trend *=* 0.004; S2 Table); and ORa optimum model, 6.09 (95% CI, 1.7–21.3; linear *P* trend *=* 0.003). By contrast, we found no association between the presence of pain and antidepressant use: ORc, 0.95 (95% CI, 0.27–3.39); ORa maximum model, 1.03 (95% CI, 0.2–4.8; S2 Table); and ORa optimum model, 0.99 (95% CI, 0.2–4.2). Details appear in Table 5.

**Table 5:** Association among presence of cancer pain, pain control, and antidepressant use.

		Antidepressant use							
		**No (n)**	**Yes (n)**	**ORc**^**a**^	**(95%**	**CI)**	**ORa opt**^**b**^	**(95%**	**CI)**
**Existence of cancer pain**									
	No	314	14	1			1		
	Yes	71	3	0.95	0.27	3.4	0.99	0.23	4.2
** **		**No (n)**	**Yes (n)**	**ORc**^**a**^	**(95%**	**CI)**	**ORa opt**^**b**^	**(95%**	**CI)**
**Pain control**									
	No pain	314	14						
	Adequate pain control	31	2	1.45	0.31	6.7	1.73	0.30	9.9
	Inadequate pain control	38	1	0.59	0.08	4.6	0.54	0.06	4.9
***Linear p trend***				0.778			0.752		

^a^ Odds ratio and 95% confidence intervals. ORc: crude odds ratio

^b^ ORa opt: adjusted OR according to the optimum model (with only selection of confounders included in the regression model according to the criteria described in Materials and Methods (*P* ≤0.20: OR 1.5–0.67, change in OR, >10%): sex, marital status, type of cancer (location), radiotherapy, and hormone therapy

When we considered joint use of anxiolytics and hypnotics, we were able to determine the associations between pain and pain control with greater precision: ORc, 3.76 (95% CI, 2.1–6.6); ORa maximum model, 4.81 (95% CI, 2.2–10.6); and ORa optimum model, 4.20 (95% CI, 2.0–8.6). Details appear in S2 and S3 Tables.

## Discussion

In our sample, 18% of patients reported cancer pain. Pain is the symptom most commonly reported by cancer patients with treatment, and it causes major suffering to patients and their families [31]. Our findings are in agreement with those of Fischer et al. [5], who found the prevalence of pain to be 20%–50% in a cancer population. However, our results are at variance with those of other studies, which reported the prevalence to be 40%, 50%, and even 90% of patients [6,22,32,33], depending on the type of cancer or disease stage. The findings of Fischer et al. [5] are used as a reference by the National Cancer Institute in their latest guidelines on cancer pain [21].

Since pain is such a widespread problem, it is particularly important to achieve effective treatment and symptom control. However, patients are often undertreated, which negatively affects their quality of life [21,33]. Complete, individualized patient assessment is therefore essential; it should address their pain history and involve a physical and psychosocial examination [20,34].

Pain control was inadequate in more than 50% of our patients. This proportion is similar to that reported by Greco et al. [3] in a review of 20 articles: control was found to be inadequate in up to 68% of patients, with a mean of 31.8%. Their data were collected from 2007 to 2013. Greco et al. found improvement in pain control with respect to a similar review published in 2008, where undertreatment was reported in up to 82% of patients [35].

In our sample, 20% of cancer patients took some type of psychotropic drug. This result is in line with the findings of Grassi et al. [36] in a review on mental disorders in cancer patients. Grassi et al. determined that 25%–30% of patients met the criteria for depression, anxiety, and insomnia (among other conditions).

Among our patients with pain, 32.4% took anxiolytics. This figure is higher than that reported by Desplenter et al. [37] in a general cancer population. However, our figure is lower than that determined by Syrowatka et al. [14] in a study of women with breast cancer; the authors found that 50.6% of patients received anxiolytics in the course of active treatment. Our figure is also lower than that reported by Barry et al. [15] among patients with metastatic cancer and pain; the authors observed anxiolytic use among 51% of patients. It should be noted that Barry et al. [15] reported that 38% of patients consumed three or more psychotropic drugs.

Although insomnia is often related to pain, only 12.2% of our patients with pain consumed hypnotics. That is because in the present study we investigated the use of hypnotics, not the presence of insomnia. Thus, it is possible that in our sample, many patients suffered from insomnia but were not receiving pharmacological treatment; some studies have reported a higher prevalence of insomnia in patients with pain [38,39]. However, that 12.2% figure is significantly higher than the proportion of patients without pain who took psychotropic drugs, which was 2.7% (*P* = 0.004).

We found that 4.1% of our patients with pain took antidepressants; that figure did not differ from the group of patients without pain. These results are much lower than those reported by Syrowatka et al. [14], Barry et al. [15], and Ng et al. [40]; those authors found the prevalence to be 22.4%, 64%, and 10.8%, respectively. However, Kierner et al. [41], who studied patients with cancer in late stages of the disease, reported that more than 75% and 90% of patients, respectively, took psychotropic and analgesic drugs. Their finding suggests that consumption may increase as the disease progresses

We found that the presence of pain and (to a greater extent) inadequate pain control were associated with a higher risk of taking anxiolytics and hypnotics, but not antidepressants. When adjusted for the optimal model, with respect to patients with no pain, the possibility (odds) of taking anxiolytics and hypnotics increased 4.74-fold and 6.09-fold, respectively, in patients with inadequate pain control. There are several explanations for this association. Lack of pain control results in poorer patient quality of life and social relationships as well as in less tolerance to cancer treatment [42]. This situation, together with the associated sleep disorders, fatigue, anxiety, and depression [42–45], could result in increased psychotropic drug use. It may also be the case, however, that certain psychotropic drugs are prescribed as analgesic adjuvants for better pain control [11,24,46].

The cross-sectional nature of our study does not permit conclusions to be drawn in the above regard. Future longitudinal studies should investigate this association in greater depth. They should determine to what extent pain leads to more psychiatric symptoms (since that is the reason for taking psychotropic drugs) and to what extent psychotropic drugs are prescribed as adjuvants in the treatment of pain, without assessing psychiatric symptoms. However, the above association suggests that if pain can be reduced—following analgesic recommendations or using interventions based on relaxation or adaptive coping strategies [24]—the patient will require fewer psychotropic drugs and obtain better quality of life.

The present study shows that despite the latest recommendations [12,13], cancer patients still have uncontrolled pain. This may be because of the following factors: the treatment and control of cancer pain is very complex; the lack of clear consensus among experts [7,9]; the need to re-evaluate the patient to ensure efficacy of treatment [7,10]; the need for individualized treatment for each patient [2]; patients’ fear of addiction; misconceptions about pain and illness [47]; ignorance about medical equipment in evaluating and managing pain; and lack of access or limited access to certain analgesics [48]. All those factors contribute to less pain control. There is therefore a need to continue research in this area.

In this study, we investigated only the presence of pain and its management and control independently of the duration. Our objective was to determine the association between pain control and psychotropic drug use. However, we believe that future studies should address any differences between acute and chronic pain.

In retrospective studies based on secondary information (records), the low quality of the information—owing to either incomplete records or lack of agreement among different records—can be a major limitation. For this reason, we selected variables that could be collected homogeneously in secondary records. However, we were unable to obtain information about symptoms of stress, anxiety, depression, and sleeping problems or identify the reasons for patients having been prescribed psychotropic drugs. We only derived information about the whole oncological situation at the time of data collection; oncological processes may, though, in some cases continue for several years.

The protocol in this study involved checking for discrepancies (contradictory information) individually with the physicians or nurses responsible for the patient; we conducted a sensitivity analysis by incorporating the information separately. We found 100% agreement regarding the analysed variables.

One limitation of this study was that the small number of patients who took antidepressants did not enable their association for inadequate pain control to be determined with sufficient precision. One of the strengths of the study was the control of confounding bias on the associations: applying the optimal model allowed the confounding bias to be minimized with the highest possible precision in the estimates.

In conclusion, we found the prevalence of pain in cancer patients to be almost 20%. Despite receiving analgesic treatment, pain was inadequately controlled in over 50% of patients. Both the presence of pain and (to a greater extent) inadequate pain control were associated with use of anxiolytics and hypnotics. This association did not appear to exist for antidepressant use.

## Supporting information

S1 Table Association between potential confounders and psychotropic drug use. (DOCX)

S2 Table Association between cancer pain,effectiveness of analgesia and anxiolytic, hynotic and antidepressant consumption adding to the maximum model. (DOCX)

S3 Table Association between existence of pain, pain control, and anxiolytic and/or hypnotic use. (DOCX)

S1 Appendix List of side effects considered in the study. (DOCX)
