# Supplementary material for: Association among presence of cancer pain, inadequate pain control, and psychotropic drug use
Source: PLoS One. 2017 Jun 8;12(6):e0178742. doi: 10.1371/journal.pone.0178742 (PMC5464574; doi:10.1371/journal.pone.0178742)
Supplement: S1 Table — (DOCX) [file pone.0178742.s001.docx]

**S1 Table. Association between potential confounders and psychotropic drug use.**

|  | | **Anxiolytic use** | | | | | **Hypnotic use** | | | | | **Antidepressant use** | | | | | **Anxiolytic and/or hypnotic use** | | | | |
| --- | --- | --- | --- | --- | --- | --- | --- | --- | --- | --- | --- | --- | --- | --- | --- | --- | --- | --- | --- | --- | --- |
|  | | **NO (n)** | **YES (n)** | **OR** | **(95%** | **CI)^a^** | **NO (n)** | **YES (n)** | **OR** | **(95%** | **CI)^a^** | **NO (n)** | **YES (n)** | **OR** | **(95%** | **CI)^a^** | **NO (n)** | **YES (n)** | **OR** | **(95%** | **CI)^a^** |
| **Gender** | |  |  |  |  |  |  |  |  |  |  |  |  |  |  |  |  |  |  |  |  |
|  | Female | 158 | 39 | 0.16 | 0.1 | 0.4 | 191 | 6 | 0.75 | 0.2 | 3.0 | 185 | 12 | 0.24 | 0.1 | 1.1 | 156 | 41 | 0.22 | 0.1 | 0.5 |
|  | Male | 126 | 5 | . | . | . | 128 | 3 | . | . | . | 129 | 2 | . | . | . | 124 | 7 | . | . | . |
| **Type of cancer (location)** |  |  |  |  |  |  |  |  |  |  |  |  |  |  |  |  |  |  |  |  |  |
|  | Breast | 72 | 26 | . | . | . | 93 | 5 | . | . | . | 89 | 9 | . | . | . | 70 | 28 | . | . | . |
|  | Rectal | 53 | 3 | 0.16 | 0.1 | 0.5 | 54 | 2 | 0.69 | 0.1 | 3.7 | 54 | 2 | 0.37 | 0.1 | 1.8 | 51 | 5 | 0.25 | 0.1 | 0.7 |
|  | Lung | 19 | 2 | 0.29 | 0.1 | 1.3 | 21 | 0 | . | . | . | 20 | 1 | 0.49 | 0.1 | 4.1 | 19 | 2 | 0.26 | 0.1 | 1.2 |
|  | B-cell lymphoma | 26 | 1 | 0.11 | 0.01 | 0.8 | 27 | 0 | . | . | . | 26 | 1 | 0.38 | 0.1 | 3.1 | 26 | 1 | 0.10 | 0.01 | 0.7 |
|  | Ovary | 9 | 3 | 0.92 | 0.2 | 3.7 | 12 | 0 | . | . | . | 12 | 0 | . | . | . | 9 | 3 | 0.83 | 0.2 | 3.3 |
|  | Others | 105 | 9 | 0.24 | 0.1 | 0.5 | 112 | 2 | 0.33 | 0.1 | 1.7 | 113 | 1 | 0.09 | 0.01 | 0.7 | 105 | 9 | 0.21 | 0.1 | 0.5 |
| **Hormone Therapy** |  |  |  |  |  |  |  |  |  |  |  |  |  |  |  |  |  |  |  |  |  |
|  | No | 243 | 34 | . | . | . | 269 | 8 | . | . | . | 269 | 8 | . | . | . | 239 | 38 | . | . | . |
|  | Yes | 41 | 10 | 1.74 | 0.8 | 3.8 | 50 | 1 | 0.67 | 0.1 | 5.5 | 45 | 6 | 4.48 | 1.5 | 13.5 | 41 | 10 | 1.53 | 0.7 | 3.3 |
| **Side Effects of Cancer Treatment** |  |  |  |  |  |  |  |  |  |  |  |  |  |  |  |  |  |  |  |  |  |
|  | No | 74 | 5 | . | . | . | 79 | 0 | . | . | . | 77 | 2 | . | . | . | 74 | 5 | . | . | . |
|  | Yes | 210 | 39 | 2.75 | 1.0 | 7.2 | 240 | 9 | 1.04 | 1.0 | 1.1 | 237 | 12 | 1.95 | 0.4 | 8.9 | 206 | 43 | 3.09 | 1.2 | 8.1 |
| **Marital Status** |  |  |  |  |  |  |  |  |  |  |  |  |  |  |  |  |  |  |  |  |  |
|  | Married | 195 | 34 | . | . | . | 222 | 7 | . | . | . | 220 | 9 | . | . | . | 193 | 36 | . | . | . |
|  | Single | 36 | 4 | 0.64 | 0.2 | 1.9 | 39 | 1 | 0.81 | 0.1 | 6.8 | 39 | 1 | 0.63 | 0.1 | 5.1 | 35 | 5 | 0.77 | 0.3 | 2.1 |
|  | Divorced/Separated | 5 | 2 | 2.29 | 0.4 | 12.3 | 7 | 0 | . | . | . | 5 | 2 | 9.78 | 1.7 | 57.4 | 5 | 2 | 2.14 | 0.4 | 11.5 |
|  | Widowed | 9 | 3 | 1.91 | 0.5 | 7.4 | 11 | 1 | 2.88 | 0.3 | 25.5 | 11 | 1 | 2.22 | 0.3 | 19.1 | 8 | 4 | 2.68 | 0.8 | 9.4 |
|  | Missing |  |  |  |  |  |  |  |  |  |  |  |  |  |  |  |  |  |  |  |  |
| **Radiotherapy** |  |  |  |  |  |  |  |  |  |  |  |  |  |  |  |  |  |  |  |  |  |
|  | No | 174 | 16 | . | . | . | 187 | 3 | . | . | . | 185 | 5 | . | . | . | 172 | 18 | . | . | . |
|  | Yes | 110 | 28 | 2.77 | 1.4 | 5.3 | 132 | 6 | 2.83 | 0.7 | 11.5 | 129 | 9 | 2.58 | 0.8 | 7.9 | 108 | 30 | 2.65 | 1.4 | 4.5 |
| **Surgery** |  |  |  |  |  |  |  |  |  |  |  |  |  |  |  |  |  |  |  |  |  |
|  | No | 148 | 19 | . | . | . | 162 | 5 | . | . | . | 162 | 5 | . | . | . | 146 | 21 | . | . | . |
|  | Yes | 136 | 25 | 1.43 | 0.8 | 2.7 | 157 | 4 | 0.83 | 0.2 | 3.1 | 152 | 9 | 1.92 | 0.6 | 5.8 | 134 | 27 | 1.40 | 0.8 | 2.6 |

^a^ Odds Ratio and 95% Confidence Intervals.
