# Supplementary material for: Association among presence of cancer pain, inadequate pain control, and psychotropic drug use
Source: PLoS One. 2017 Jun 8;12(6):e0178742. doi: 10.1371/journal.pone.0178742 (PMC5464574; doi:10.1371/journal.pone.0178742)
Supplement: S2 Table — (DOCX) [file pone.0178742.s002.docx]

|  | **ANXIOLYTIC USE** | | | | | **HYPNOTIC USE** | | | | | **ANTIDEPRESSANT USE** | | | | |
| --- | --- | --- | --- | --- | --- | --- | --- | --- | --- | --- | --- | --- | --- | --- | --- |
|  | **NO (n)** | **YES (n)** | **ORa^a^** | **(95%** | **CI)** | **NO (n)** | **YES (n)** | **ORa^a^** | **(95%** | **CI)** | **NO (n)** | **YES (n)** | **ORa^a^** | **(95%** | **CI)** |
| **Existence of cancer pain** |  |  |  |  |  |  |  |  |  |  |  |  |  |  |  |
| No | 284 | 44 | 1 |  |  | 319 | 9 | 1 |  |  | 314 | 14 | 1 |  |  |
| Yes | 50 | 24 | 3.64 | 1.6 | 8.4 | 65 | 9 | 5.57 | 1.6 | 19.0 | 71 | 3 | 1.03 | 0.2 | 4.8 |
|  | **NO (n)** | **YES (n)** | **ORa^a^** | **(95%** | **CI)** | **NO (n)** | **YES (n)** | **ORa^a^** | **(95%** | **CI)** | **NO (n)** | **YES (n)** | **ORa^a^** | **(95%** | **CI)** |
| **Pain control** |  |  |  |  |  |  |  |  |  |  |  |  |  |  |  |
| No pain | 284 | 44 | 1 |  |  | 319 | 9 | 1 |  |  | 314 | 14 |  |  |  |
| Adequate pain control | 25 | 8 | 2.46 | 0.8 | 7.8 | 29 | 4 | 3.73 | 0.7 | 19.2 | 31 | 2 | 1.99 | 0.3 | 14.0 |
| Inadequate pain control | 24 | 15 | 4.98 | 1.8 | 13.7 | 34 | 5 | 7.60 | 1.8 | 31.3 | 38 | 1 | 0.58 | 0.1 | 5.5 |
| ***Linear p trend*** |  |  | 0.001 |  |  |  |  | 0.004 |  |  |  |  | 0.807 |  |  |

**S2 Table. Association between cancer pain, effectiveness of analgesia and anxiolytic, hynotic and antidepressant consumption adding to the maximum model.**

^a^ Odds Ratio and 95% Confidence Intervals. OR denotes "adjusted" OR adding to the maximum model (for all potentially confounding variables): Gender, Age, Maritas Status, Children, Education Level, Cancer Diagnosis, Cancer Therapy (Chemotherapy, Radiotherapy, Hormone Therapy, Biological Therapy and Surgery), Side Effects of Cancer Treatment and Fatigue.
