# Supplementary material for: Association among presence of cancer pain, inadequate pain control, and psychotropic drug use
Source: PLoS One. 2017 Jun 8;12(6):e0178742. doi: 10.1371/journal.pone.0178742 (PMC5464574; doi:10.1371/journal.pone.0178742)
Supplement: S3 Table — (DOCX) [file pone.0178742.s003.docx]

**S3 Table. Association between existence of pain, pain control, and anxiolytic and/or hypnotic use.**

|  |  | **Anxiolytic or hypnotic use** | | | |  |  | |  |  | |  |
| --- | --- | --- | --- | --- | --- | --- | --- | --- | --- | --- | --- | --- |
|  |  | **NO (n)** | **YES (n)** | **ORc^a^** | **(95%** | **CI)** | **ORa máx^b^** | **(95%** | **CI)** | **ORa opt^c^** | **(95%** | **CI)** |
| **Existence of cancer pain** |  |  |  |  |  |  |  |  |  |  |  |  |
|  | No | 280 | 48 | 1 |  |  | 1 |  |  | 1 |  |  |
|  | Yes | 45 | 29 | 3.76 | 2.1 | 6.6 | 4.81 | 2.2 | 10.6 | 4.20 | 2.0 | 8.6 |
|  |  | **NO (n)** | **YES (n)** | **ORc^a^** | **(95%** | **CI)** | **ORa máx^b^** | **(95%** | **CI)** | **ORa opt^c^** | **(95%** | **CI)** |
| **Pain control** |  |  |  |  |  |  |  |  |  |  |  |  |
|  | No pain | 280 | 48 |  |  |  |  |  |  |  |  |  |
|  | Adequate pain control | 23 | 10 | 2.54 | 1.1 | 5.7 | 3.14 | 1.1 | 9.1 | 2.51 | 0.9 | 6.7 |
|  | Inadequate pain control | 21 | 18 | 5.00 | 2.5 | 10.1 | 6.95 | 2.6 | 18.2 | 6.52 | 2.7 | 15.9 |
| ***Linear p trend*** |  |  |  | <0.001 |  |  | <0.001 |  |  | <0.001 |  |  |

^a^ Odds Ratio and 95% Confidence Intervals. ORc denotes “crude Odds ratio”.

^b^ ORa max: adjusted OR according to the maximum model (with all the potential confounders in the regression model): age, sex, marital status, education level, having children, type of cancer (location), chemotherapy, radiotherapy, hormone therapy, biological therapy, surgery, side effects of cancer treatment, and fatigue.

^c^ ORa opt: adjusted OR according to the optimum model (with only a selection of confounders included in the regression model according to the criteria described in methods (*p* ≤ 0.20, 1.5 < OR < 0.67, change in OR > 10%): sex, marital status, type of cancer (location), and side effects of cancer treatment.
