# Supplementary material for: Association among presence of cancer pain, inadequate pain control, and psychotropic drug use
Source: PLoS One. 2017 Jun 8;12(6):e0178742. doi: 10.1371/journal.pone.0178742 (PMC5464574; doi:10.1371/journal.pone.0178742)
Supplement: S1 Appendix — (DOCX) [file pone.0178742.s004.docx]

**S1 Appendix. List of side effects considered in the study.**

The symptoms considered as side effects are those established by the National Cancer Institute*.

Anemia

Appetite Loss

Bleeding and Bruising (Thrombocytopenia)

Constipation

Delirium

Diarrhea

Edema

Fatigue

Hair Loss (Alopecia)

Infection and Neutropenia

Lymphedema

Memory or Concentration Problems

Mouth and Throat Problems

Nausea and Vomiting

Nerve Problems (Peripheral Neuropathy)

Pain

Sexual and Fertility Problems (Men)

Sexual and Fertility Problems (Women)

Skin and Nail Changes

Sleep Problems

Urinary and Bladder Problems

*National Cancer Institute. Side Effects. 2015 Apr 19 [cited 28 November 2016]. In: National Cancer Institute Web [Internet]. Bethesda. Available from: <https://www.cancer.gov/about-cancer/treatment/side-effects>
